# The Role of Molecular Testing in Pediatric Meningitis Surveillance in Southern and East African Countries, 2008–2017

**DOI:** 10.1093/infdis/jiab092

**Published:** 2021-09-01

**Authors:** Mignon du Plessis, Linda de Gouveia, Cesar Freitas, Negga Asamene Abera, Budiaki Sylvie Lula, Julia Liliane Raboba, Aquino Albino Nhantumbo, Elana Jantjies, Jeannine Uwimana, Nomcebo Phungwayo, Gugu Maphalala, Gilbert Masona, John Muyombe, David Mugisha, Esther Nalumansi, Moses Odongkara, Chileshe Lukwesa-Musyani, Ruth Nakazwe, Vongai Dondo, John Macharaga, Goitom G Weldegebriel, Jason M Mwenda, Fatima Serhan, Adam L Cohen, Fernanda C Lessa, Anne von Gottberg

**Affiliations:** 1 Centre for Respiratory Diseases and Meningitis, National Institute for Communicable Diseases, National Health Laboratory Service, Johannesburg, South Africa; 2 School of Pathology, Faculty of Health Sciences, University of the Witwatersrand, Johannesburg, South Africa; 3 Hospital Pediatrico David Bernardino, Luanda, Angola; 4 Bacteriology National Reference Laboratory, Ethiopian Public Health Institute, Addis Ababa, Ethiopia; 5 Department of Microbiology National Reference Laboratory, Ministry of Health, Maseru, Lesotho; 6 Department of Child Health, Teaching Hospital, Centre Hospitalier Universitaire Mère Enfant Tsaralàlana, Antananarivo, Madagascar; 7 Instituto Nacional de Saúde, Maputo, Mozambique; 8 Namibia Institute of Pathology, Microbiology, and Windhoek Central Reference Laboratory, Windhoek, Namibia; 9 Centre Hospitalier Universitaire de Kigali, Kigali, Rwanda; 10 National Surveillance Laboratory, eSwatini Health Laboratory Services, eSwatini; 11 Bacteriology Laboratory, Bugando Medical Centre, Mwanza, United Republic of Tanzania; 12 Ministry of Health, Bacteriology Laboratory, Mulago Teaching Hospital, Uganda; 13 Ministry of Health, University Teaching Hospital, Pathology and Microbiology Department, Lusaka, Zambia; 14 Harare Children’s Hospital, Harare, Zimbabwe; 15 World Health Organization Regional Office for Africa, Inter-Country Support Team, Harare, Zimbabwe; 16 World Health Organization Regional Office for Africa, Brazzaville, Republic of Congo; 17 World Health Organization, Geneva, Switzerland; 18 Division of Bacterial Diseases, National Center for Immunization and Respiratory Diseases, Centers for Disease Control and Prevention, Atlanta, Georgia, USA

**Keywords:** pediatric bacterial meningitis surveillance, Africa, real-time PCR, molecular testing, IB-VPD, meningitis pathogens

## Abstract

**Background:**

As part of the global Invasive Bacterial Vaccine-Preventable Diseases Surveillance Network, 12 African countries referred cerebrospinal fluid (CSF) samples to South Africa’s regional reference laboratory. We evaluated the utility of real-time polymerase chain reaction (PCR) in detecting and serotyping/grouping *Haemophilus influenzae*, *Neisseria meningitidis,* and *Streptococcus pneumoniae* (HNS).

**Methods:**

From 2008 to 2017, CSF samples collected from children <5 years old with suspected meningitis underwent routine microbiology testing in-country, and 11 680 samples were submitted for HNS PCR at the regional reference laboratory. Unconditional logistic regression, with adjustment for geographic location, was performed to identify factors associated with PCR positivity.

**Results:**

The overall HNS PCR positivity rate for all countries was 10% (1195 of 11 626 samples). In samples with both PCR and culture results, HNS PCR positivity was 11% (744 of 6747 samples), and HNS culture positivity was 3% (207 of 6747). Molecular serotype/serogroup was assigned in 75% of PCR-positive specimens (762 of 1016). Compared with PCR-negative CSF samples, PCR-positive samples were more often turbid (adjusted odds ratio, 6.80; 95% confidence interval, 5.67–8.17) and xanthochromic (1.72; 1.29–2.28), had elevated white blood cell counts (6.13; 4.71–7.99) and high protein concentrations (5.80; 4.34–7.75), and were more often HNS culture positive (32.70; 23.18–46.12).

**Conclusion:**

PCR increased detection of vaccine-preventable bacterial meningitis in countries where confirmation of suspected meningitis cases is impeded by limited culture capacity.

Bacterial meningitis, commonly caused by *Haemophilus influenzae, Neisseria meningitidis,* and *Streptococcus pneumoniae,* is responsible for significant disease and death, particularly in children aged <5 years. In this age group, high rates of *H. influenzae* type b and *S. pneumoniae* disease and death occur in sub-Saharan Africa [1]. The highest incidence of meningococcal disease occurs in the African meningitis belt and is characterized by periodic epidemics, with incidence rates approaching 1000 per 100 000 population [2]. During the last 20 years, several initiatives have supported the introduction of *H. influenzae* type b, meningococcal, and pneumococcal conjugate vaccines (PCVs) in several African countries [3–5].

Advocacy for vaccine introduction and monitoring of vaccine impact is reliant on robust and accurate surveillance data, which are lacking in many low-income countries. The Paediatric Bacterial Meningitis (PBM) Surveillance network was launched by the World Health Organization (WHO) Regional Office for Africa in 2001 to provide data on clinical, epidemiological, and laboratory-confirmed bacterial meningitis in children aged <5 years [6]. In 2008, PBM was incorporated into a larger global network conducting sentinel hospital surveillance for invasive bacterial vaccine-preventable diseases as part of the WHO-coordinated Global Invasive Bacterial Vaccine-Preventable Disease (IB-VPD) Surveillance Network [7]. The objectives were to strengthen existing surveillance platforms and standardize methods to accurately describe etiology, disease burden, and epidemiology. Moreover, data are used to monitor *H. influenzae* type b and pneumococcal vaccine impact in countries that have already introduced vaccines and to identify appropriate pneumococcal vaccines for those countries yet to introduce these vaccines.

Clinical management of acute bacterial meningitis is usually empirical; however, etiology is important in guiding prevention through appropriate vaccination and assisting with outbreak management. Diagnosis historically depends on cerebrospinal fluid (CSF) examination and bacterial culture; the latter remains the reference standard for pathogen detection because of its specificity. Many African microbiology laboratories lack resources and capacity to reliably confirm etiology or perform characterization of pathogens [8]. Polymerase chain reaction (PCR) has the advantage of faster turnaround time and improved sensitivity, particularly when Gram stain or culture results are negative [9, 10]. However, stringent quality control procedures are required to avoid false-positive PCR results that may occur because of contamination. False-negative results may occur as a result of suboptimal sample collection, storage, DNA extraction, or inhibitors that may be present in the sample or added as a result of the extraction process [11]. Furthermore, real-time PCR cycle threshold (C_t_) cutoff values for positivity differ between laboratories. Consideration should be given to the purpose of the test and whether emphasis should be on sensitivity or specificity. Diagnostic sensitivity should be prioritized for diseases that are treatable and for which there are public health implications [11].

As a WHO-designated regional reference laboratory (RRL) for the southern African region of the Global IB-VPD Surveillance Network, the National Institute for Communicable Diseases (NICD) in South Africa conducts PCR testing and serotyping/serogrouping for *H. influenzae, N. meningitidis,* and *S. pneumoniae* on CSF samples collected from children aged <5 years with suspected bacterial meningitis. We describe the utility of PCR in determining the prevalence and serotype/serogroup of these 3 pathogens in CSF specimens from 12 southern and east African countries, from 2008 through 2017.

## METHODS

### Case Definition

A suspected case was defined as sudden onset of fever (>38.5ºC rectal or 38ºC axillary) and 1 of the following signs: neck stiffness, altered consciousness in the absence of an alternative diagnosis, or other meningeal signs in children aged 0–59 months admitted to a sentinel hospital conducting surveillance [12, 13]. A confirmed case of meningitis was defined as the isolation of *H. influenzae, N. meningitidis,* or *S. pneumoniae* or detection of any of the 3 bacteria by antigen detection or PCR, from CSF sample from a child with clinical symptoms suggestive of bacterial meningitis.

### Surveillance Sites

Participating countries included Angola, Ethiopia, Lesotho, Madagascar, Mozambique, Namibia, Rwanda, eSwatini (formally Swaziland), Tanzania, Uganda, Zambia, and Zimbabwe. Ethiopia has 3 surveillance sites and all are located in the “meningitis belt.” For inclusion in this analysis, the sites had to have performed continuous surveillance (and sample collection) for at least 10 months of each reporting year. Site selection was based on access to pediatric patients, capacity to perform lumbar puncture and microbiology testing, and willingness to share samples and data. WHO and NICD provided training and site assessments, and sites participated in the United Kingdom National External Quality Assessment Services program.

### Hospital Laboratory Testing

CSF samples were collected from suspected cases and tested at the sentinel site microbiology or chemistry laboratory in-country, using ≥1 of the following: bacterial culture, Gram stain, rapid diagnostic tests (BinaxNOW for *S. pneumoniae* or bacterial antigen rapid latex agglutination test for other pathogens), protein concentration, and cell count. CSF appearance was also assessed. Regardless of phenotypic test results, residual CSF specimens were stored at −20ºC or −70ºC in-country. Samples were sent periodically, using the same courier service, frozen on ice packs, to the RRL in South Africa for molecular testing for surveillance and quality control purposes. Phenotypic laboratory results (as reported by site) were not verified at the RRL.

### Reference Laboratory Molecular Testing

Total nucleic acid was extracted from 200 µL of CSF using the Roche MagNA Pure 96 instrument and Viral NA small volume kit (Roche). Samples were tested in duplicate using a multiplex, real-time PCR assay targeting *hpd* (*H. influenzae*), *ctrA* (*N. meningitidis*), and *lytA* (*S. pneumoniae*) genes (HNS assay) [14] and an ABI 7500 Fast real-time PCR instrument (Applied Biosystems). Positivity was assigned based on target amplification in both duplicates (C_t_, <40). Real-time PCR, detecting the human RNase P (RNP) gene [15], was performed to confirm sample integrity and the absence of inhibitors—standard practice for all clinical specimens processed at the RRL. PCR results for clinical samples that test negative for the targeted pathogen and with C_t_ values ≥36 for RNP are routinely reported as inconclusive because they may be falsely negative. The RRL is accredited by the South African National Accreditation System, and the workflow and assays are conducted using standardized procedures with systems in place to detect and minimize errors and the risk of DNA contamination.

### Reference Laboratory Molecular Serotyping/Serogrouping

Real-time PCR serotyping/serogrouping was performed on samples that tested HNS PCR positive. CSF samples positive for *hpd* or *ctrA* targets (C_t_, <40) underwent PCR serotyping/grouping. *H. influenzae* serotyping was carried out using 3 multiplex reactions incorporating *igA* as a second *H. influenzae* confirmatory target, *bexA* (involved in capsule transport), and serotype-specific genes for serotypes a to f [16, 17]. *N. meningitidis* serogrouping was performed using 2 multiplex reactions for detection of serogroups A, W, X and B, C, Y [14]. Pneumococcal serotyping was conducted on *lytA*-positive samples with C_t_ values ≤35, as described elsewhere [18–20]. Serotype 8 was included as an additional reaction and combined with the detection of serotype 19A [21] (Supplementary Table 1). Samples that were PCR negative for the 38 serotypes were recorded as “Neg38.”

### Data Analysis

Positive and negative results with HNS PCR were compared with CSF characteristics and phenotypic laboratory results, using unconditional logistic regression, controlling for geographic location (country), performed with Stata software, version 14 (StataCorp). Differences were considered significant at *P* < .05.

### Ethics Review and Approval

The WHO Regional Office for Africa Ethics Review Committee granted a waiver indicating that collection of CSF specimens from children admitted with meningitis, to test for and monitor circulation of bacterial pathogens before and after introduction of vaccines, is part of routine surveillance for vaccine-preventable diseases.

## RESULTS

**Figure 1.**
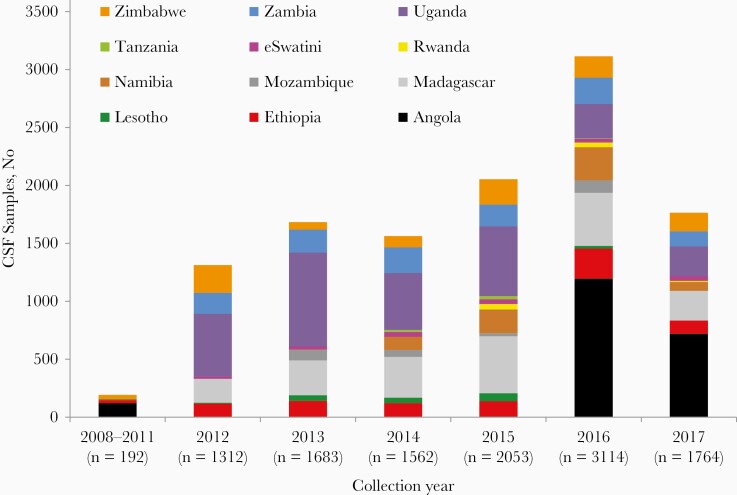
Numbers of cerebrospinal fluid (CSF) samples, by country and year, submitted for polymerase chain reaction detection of *Streptococcus pneumoniae*, *Neisseria meningitidis,* and *Haemophilus influenzae* to the Global Invasive Bacterial Vaccine-Preventable Diseases Surveillance Network regional reference laboratory in South Africa, 2008–2017 (N = 11 680).

A total of 11 680 CSF samples, collected from 2008 through 2017, were received for testing at the RRL; most samples were collected from 2012 onward (Figure 1). Countries submitting samples included Angola (n = 2042), Ethiopia (n = 911), Lesotho (n = 201), Madagascar (n = 2067), Mozambique (n = 285), Namibia (n = 683), Rwanda (n = 97), eSwatini (n = 197), Tanzania (n = 51), Uganda (n = 2998), Zambia (n = 1148), and Zimbabwe (n = 1000) (Supplementary Table 2). Forty-one CSF samples were excluded from testing because the tubes were empty, leaving a total of 11 639 samples to be tested. Matched cultures were received for 42 CSF samples, and identification was confirmed at the RRL.

The overall HNS PCR positivity rate was 10% (1195 of 11 626 samples), of which 2% (242 of 11 626) were *hpd* (*H. influenzae*) positive, 1.5% (170 of 11 626) were *ctrA* (*N. meningitidis*) positive, and 7% (783 of 11 626) were *lytA* (*S. pneumoniae*) positive (Table 1). Among samples with RNP C_t_ values <36, the detection rate was 12% (909 of 7314 samples): 2.7% (199 of 7314) for *H. influenzae,* 1.8% (130 of 7314) for *N. meningitidis,* and 8% (580 of 7314) for *S. pneumoniae*. Among samples that were HNS PCR negative, 37% (3894 of 10 431 samples) had RNP C_t_ values ≥36, indicative of low or undetectable levels of human DNA. Among samples that were HNS PCR positive in which RNP PCR had been done, 18% (197 of 1115) had low or undetectable levels of human DNA.

**Table 1.** Polymerase Chain Reaction Positivity Rate and Serotype/Group Assignment for *Haemophilus influenzae, Neisseria meningitidis,* and *Streptococcus pneumoniae* in Children <5 Years Old in Southern and East Africa, 2008–2017^a^

Outcome	CSF Samples by Collection Year, No. (%)b										
	2008	2009	2010	2011	2012	2013	2014	2015	2016	2017	Total
Total no.	7	5	124	56	1312	1683	1562	2053	3103	1734	11 639
*H. influenzae* positive	0	1 (20)	9 (7)	2 (4)	33 (3)	38 (2)	46 (2)	37 (1.8)	49 (1.6)	27 (1.6)	242 (2)
Serotypec	NA	0 (0)	7/9 (78)	2/2 (100)	16/33 (48)	19/38 (50)	19/46 (41)	17/37 (46)	28/49 (57)	17/27 (63)	123/242 (51)
*N. meningitidis* positive	0	2/5 (40)	6 (5)	2 (4)	11 (0.8)	26 (1.5)	41 (3)	36 (1.8)	27 (0.9)	19 (1)	170 (1.5)
Serogroup W^d^	NA	0 (0)	1/6 (17)	2/2 (100)	9/11 (82)	21/26 (81)	34/41 (83)	32/36 (89)	7/27 (26)	9/19 (47)	115/170 (68)
*S. pneumoniae* positive	2 (29)	0	23 (19)	9 (16)	165 (13)	188 (11)	103 (7)	108 (5)	127 (4)	58 (3)	783 (7)
PCV13 serotype^e,f^	1/2 (50)	NA	18/22 (82)	4/4 (100)	69/117 (59)	90/137 (66)	36/85 (42)	36/85 (42)	48/100 (48)	12/46 (26)	316/604 (52)
PCR dual target^g^	0	0	0	0	4 (0.3)	6 (0.4)	3 (0.2)	0	0	0	13 (0.1)
PCR negative	2 (29)	1 (20)	72 (58)	19 (34)	612 (47)	810 (48)	951 (61)	1414 (69)	1757 (57)	902 (52)	6540 (56)
RNase P negative	3 (43)	1 (20)	14 (11)	24 (43)	487 (36)	615 (37)	418 (27)	458 (22)	1143 (37)	731 (42)	3894 (33)

Abbreviations: CSF, cerebrospinal fluid; *H. influenzae, Haemophilus influenzae*; *N. meningitidis, Neisseria meningitidis*; NA, not applicable; PCR, polymerase chain reaction; PCV13, 13-valent pneumococcal conjugate vaccine; *S. pneumoniae, Streptococcus pneumoniae*.

^a^Findings from the Global Invasive Bacterial Vaccine-Preventable “Disease Surveillance Network. Participating countries include Angola, Ethiopia, Lesotho, Madagascar, Mozambique, Namibia, Rwanda, eSwatini (Swaziland), Tanzania, Uganda, Zambia, and Zimbabwe

bColumn percentages are no. divided by “Total no.’’ so these are column percentages, except where x/y is shown.

^c^Denominators represent *H. influenzae*–positive samples.

^d^Denominators represent *N. meningitidis*–positive samples.

^e^*LytA*-positive samples with cycle threshold (C_t_) values ≥36 (179 of 783 [23%]) were excluded from PCR serotyping [18].

^f^Denominators represent *S. pneumoniae*–positive samples.

^g^Dual target detections included the following: *H. influenzae* + *S. pneumoniae* (n = 7), *N. meningitidis* + *S. pneumoniae* (n = 4), and *H. influenzae* + *N. meningitidis* (n = 2).

Molecular serotype (for *H. influenzae* and *S. pneumoniae*) or serogroup (for *N. meningitidis*) was assigned in 75% of HNS PCR–positive samples (762 of 1016): 69% (166 of 242) for *H. influenzae,* 93% (158 of 170) for *N. meningitidis,* and 73% (438 of 604) for *S. pneumoniae* (179 samples had *lytA* C_t_ values >35 and were excluded from serotyping). For *hpd*-positive (*H. influenzae*) samples, 5% (4 of 79) with high *hpd* C_t_ values (36–39) had a serotype assigned, whereas 56% of CSF samples (15 of 27) with high *ctrA* (*N. meningitidis*) C_t_ values (36–39) had a serogroup assigned by PCR.

In samples with both PCR and culture results, HNS PCR positivity was 11% (744 of 6747 samples), whereas HNS culture positivity was 3% (207 of 6747). Among these 744 PCR-positive samples, 21% (155 of 744) were reported to be culture positive for their matching pathogen: 10% (15 of 147), 14% (13 of 93), and 25% (127 of 504) for *H. influenzae, N. meningitidis,* and *S. pneumoniae,* respectively (Table 2). Bacteria other than concordant *H. influenzae, N. meningitidis,* or *S. pneumoniae* were reported from 4% of HNS PCR–positive samples (31 of 744). Two CSF samples that were reported as culture positive for *S. pneumoniae* were PCR positive only for *N. meningitidis* (serogroups A and W), and 2 CSF samples reported as culture positive for *H. influenzae* were PCR positive only for *S. pneumoniae* (serotypes 4 and 12ABF/44/46). In 2 CSF samples reported as culture positive for *N. meningitidis,* 1 was PCR positive only for *S. pneumoniae* (7A/F), and the other was PCR positive for *N. meningitidis* (W) and *S. pneumoniae* (6A/6B).

**Table 2.** Bacterial Culture Results in Cerebrospinal Fluid Samples Polymerase Chain Reaction Positive for *Haemophilus influenzae* (n = 242), *Neisseria meningitidis* (n = 170), or *Streptococcus pneumoniae* (n = 783) in Children <5 Years Old in Southern and East Africa, 2008–2017^a^

PCR Target Gene (Pathogen)	CSF Samples, No./Total (%)			
	Culture Positive		Culture Negative (No Growth)	No Culture Data (Missing/Unknown)
	Matching Pathogen	Other Bacteria		
*hpd* (*H. influenzae*)	15/147 (10)	8/147 (5)^b^	124/147 (84)	95/242 (39)
*ctrA* (*N. meningitidis*)	13/93 (14)	2/93 (2)^c^	78/93 (84)	77/170 (45)
*lytA* (*S. pneumoniae*)	127/504 (25)	21/504 (3)^d^	356/504 (71)	279/783 (36)
Total	155/744 (21)	31/744 (4)	558/744 (75)	451/1195 (38)

Abbreviations: CSF, cerebrospinal fluid; *H. influenzae, Haemophilus influenzae*; *N. meningitidis, Neisseria meningitidis*; PCR, polymerase chain reaction; *S. pneumoniae, Streptococcus pneumoniae*.

^a^Findings from the Global Invasive Bacterial Vaccine-Preventable Disease Surveillance Network.

^b^Including Enterobacteriaeae (n = 2), *Streptococcus agalactiae* (n = 1), *Staphylococcus* spp. (n = 1), and unspecified (n = 4).

^c^*S. pneumoniae* (n = 2).

^d^Including Enterobacteriaeae (n = 3), *H. influenzae* (n = 2), *N. meningitidis* (n = 1), *Streptococcus* spp. (n = 6), and *Staphylococcus* spp. (n = 1), unspecified (n = 8).

Among samples that tested HNS PCR negative (with reported culture results), 0.8% (47 of 6003) were reported as culture positive for *H. influenzae* (n = 6), *N. meningitidis* (n = 1), or *S. pneumoniae* (n = 40). Among HNS culture–positive samples, PCR concordance for the matched pathogen was 65% (15 of 23) for *H. influenzae,* 93% (14 of 15) for *N. meningitidis,* and 75% (127 of 169) for *S. pneumoniae*.

In addition to the 1195 single-pathogen positives, 13 samples were PCR positive for 2 targets: *hpd* (*H. influenzae*) and *lytA* (*S. pneumoniae*) (n = 7), *ctrA* (*N. meningitidis*) and *lytA* (*S. pneumoniae*) (n = 4), and *hpd* (*H. influenzae*) and *ctrA* (*N. meningitidis*) (n = 2) (Supplementary Table 3). One of these samples (positive for both *lytA* and *ctrA* genes) was culture positive for *N. meningitidis* with a positive Gram stain result (gram-negative cocci) and was PCR positive for *N. meningitidis* serogroup W and *S. pneumoniae* serotype 6A/6B. Six of these samples were reported as culture negative with no bacteria observed on the Gram stain, and 6 samples had no recorded phenotypic laboratory data.

Among HNS PCR–positive samples (n = 1195), for which routine microbiology testing (white blood cell count, protein concentration, culture, rapid test, Gram stain) was performed, 53% (477 of 897), 70% (254 of 364), 76% (558 of 738), 32% (65 of 202), and 58% (341 of 591) yielded negative results, respectively (Table 3). Seventeen samples were negative with all 5 tests. Where data were available, we compared phenotypic results and CSF parameters with PCR results (Table 3).

**Table 3.** Cerebrospinal Fluid Characteristics Associated With HNS Polymerase Chain Reaction Positive Results in Children <5 Years Old in Southern and East Africa, 2008–2017^a^

Characteristic	CSF Samples, No. (%)^b^						aOR^c^ (95% CI)	*P* Value
	HNS PCR Positive			HNS PCR Negative				
	Total (n = 1195)	C_t_ ≤35 (n = 910)	C_t_ 36–39 (n = 285)	Total (n= 10 299)	RNase P PCR C_t_ ≤35 (n = 6405)	RNase P PCR C_t_ ≥36 (n = 3894)		
CSF appearance								
Clear	437 (52)	277 (43)	160 (79)	5319 (76)	3128 (72)	2191 (84)	Reference	…
Turbid	275 (33)	258 (40)	17 (8)	514 (7)	400 (9)	114 (4)	6.80 (5.67–8.17)	<.001
Bloody	63 (8)	55 (9)	8 (4)	613 (9)	440 (10)	173 (7)	1.26 (.95–1.67)	.11
Xanthochromic	65 (8)	48 (8)	17 (8)	527 (8)	382 (9)	145 (6)	1.72 (1.29–2.28)	<.001
Unknown	355 (30)	272 (30)	83 (29)	3326 (32)	2055 (32)	1271 (33)	…	…
WBC count,								
<10/μL	477 (53)	298 (44)	179 (81)	6415 (84)	3756 (82)	2659 (90)	Reference	…
10–100/μL	317 (35)	284 (42)	33 (15)	908 (12)	674 (15)	234 (8)	5.36 (4.53–6.33)	<.001
>100/μL	103 (11)	95 (14)	8 (4)	212 (3)	164 (4)	48 (2)	6.13 (4.71–7.99)	<.001
Unknown	298 (25)	233 (26)	65 (23)	2764 (27)	1811 (28)	953 (24)	…	…
Protein level								
≤100 mg/dL	254 (70)	190 (65)	64 (88)	3768 (91)	2294 (90)	1474 (92)	Reference	…
>100 mg/dL	110 (30)	101 (35)	9 (12)	383 (9)	257 (10)	126 (8)	5.80 (4.34–7.75)	<.001
Unknown	831 (70)	619 (68)	212 (74)	6148 (60)	3854 (60)	2294 (59)	-	…
Culture								
*H. influenzae,S. pneumoniae,* or *N. meningitidis*	160 (22)	159 (29)	1 (0.5)	43 (0.7)	26 (1)	17 (0.7)	32.70 (23.18–46.12)	<.001
Other bacteria	20 (3)	15 (3)	5 (3)	124 (2)	76 (2)	48 (2)	1.42 (.86–2.33)	.17
Negative	558 (76)	375 (68)	183 (97)	5759 (97)	3496 (97)	2263 (97)	Reference	…
Unknown	457 (38)	361 (40)	96 (34)	4373 (42)	2807 (44)	1566 (40)	…	…
Rapid diagnostic test								
*H. influenzae,S. pneumoniae* or *N. meningitidis*	136 (67)	128 (78)	8 (22)	504 (24)	321 (25)	183 (21)	15.87 (10.23–24.63)	<.001
Other bacteria	1 (0.5)	1 (0.6)	0 (0)	14 (0.7)	11 (1)	3 (0.3)	1.25 (.12–12.86)	.85
Negative	65 (32)	36 (22)	29 (78)	1626 (76)	939 (74)	687 (79)	Reference	…
Unknown	993 (83)	745 (82)	248 (87)	8155 (79)	5134 (80)	3021 (78)	…	…
Gram stain								
Gram-positive cocci	154 (26)	151 (34)	3 (2)	186 (4)	116 (5)	70 (4)	7.94 (6.18–10.19)	<.001
Gram-negative bacilli/coccobacilli	73 (12)	53 (12)	20 (13)	472 (11)	252 (10)	220 (12)	1.02 (.74–1.39)	.92
Gram-negative cocci	23 (4)	21 (5)	2 (1)	77 (2)	25 (1)	52 (3)	3.64 (2.16–6.13)	<.001
Negative or nothing of significance	341 (58)	212 (48)	129 (84)	3604 (83)	2166 (84)	1438 (81)	Reference	…
Unknown	604 (51)	473 (52)	131 (46)	5960 (58)	3846 (60)	2114 (54)	…	…

Abbreviations: aOR, adjusted odds ratio; CI, confidence interval; CSF, cerebrospinal fluid; C_t_, cycle threshold; HNS, multiplex PCR that detects *H. influenzae*, *N. meningitidis,* and *S. pneumoniae*; PCR, polymerase chain reaction; WBC, white blood cell.

^a^Findings from the Global Invasive Bacterial Vaccine-Preventable Disease Surveillance Network.

^b^Denominators used to calculate percentages for each characteristic excluded those samples with data unknown.

^c^aORs, controlling for geographic location (country), comparing characteristics between HNS PCR–positive and HNS PCR–negative samples.

Compared with CSF samples from individuals who were HNS PCR negative, samples from individuals who were HNS PCR positive were more likely to be turbid (adjusted odds ratio, 6.80; 95% confidence interval, 5.67–8.17), xanthochromic (1.72; 1.29–2.28), and have an elevated white blood cell count >100 (6.13; 4.71–7.99) and a protein concentration >100 mg/dL (5.80; 4.34–7.75). They were also more often positive at culture for *H. influenzae, S. pneumoniae,* or *N. meningitidis* (adjusted odds ratio, 32.70; 95% confidence interval, 23.18–46.12), rapid diagnostic test for *H. influenzae, S. pneumoniae,* or *N. meningitidis* (15.87; 10.23–24.63), and Gram stain (7.94 [6.18–10.19] for gram-positive and 3.64 [2.16–6.13]) for gram-negative cocci). We observed similar results when applying the logistic regression analysis to include only those samples with RNP C_t_ values <36, when comparing HNS C_t_ values >36 versus ≤36, and when comparing RNP C_t_ values >36 versus ≤36 (Supplementary Table 4*A*–4*C*).

## Discussion

Identification of pathogens causing acute bacterial meningitis provides important epidemiological data to guide treatment and to support decisions on vaccine introduction and impact monitoring. Surveillance in low-income countries underestimates the prevalence of bacterial meningitis because of the low sensitivity of culture, lack of resources and training for bacterial culture, and limited access to care. In addition, many African countries lack capacity to do molecular testing, which is particularly useful for patients receiving antibiotic treatment at the time of specimen collection. In this analysis, PCR increased the detection of laboratory-confirmed meningitis almost 4-fold compared with culture. Regardless of the country where CSF was collected, samples with xanthochromic or turbid appearance, elevated white blood cell count, or high protein levels were more likely to be PCR positive than samples without these characteristics. In addition, molecular typing assigned a serotype/group in 75% of HNS PCR–positive samples, which has been helpful for evaluating the impact of PCVs in African countries participating in WHO’s Global IB-VPD Surveillance Network [22].

The value of PCR in determining bacterial meningitis disease burden has been previously demonstrated in low-resource settings. Detection rates for culture and PCR were higher in these earlier studies than those detected in our study; this may be influenced by differences in case definitions and sampling, timing of PCV introduction, and the fact that testing was performed at hospital-site laboratories. In 2002–2008, the PBM network reported a 7% culture positivity rate among 22 participating African countries [6].

In Ethiopia, shortly after the introduction of 10-valent PCVs in 2011, HNS PCR was performed on turbid CSF samples collected from patients with suspected bacterial meningitis of all ages at 3 referral hospitals in 2012–2013. The positivity rate was 11% for culture and 33% for real-time PCR [23]. Similarly, in Mozambique, a year after PCV introduction in 2012, CSF samples from children with suspected bacterial meningitis at 3 regional hospitals had a significantly higher PCR positivity rate of 48%, versus 7% for culture for *H. influenzae, N. meningitidis,* and *S. pneumoniae* [24]. In São Paulo, Brazil, in 2007–2009, PCR increased the pathogen detection rate in culture-negative CSF and serum samples by 20% (*H. influenzae*), 85% (*N. meningitidis*), and 52% (*S. pneumoniae*), leading to the successful introduction of PCR into bacterial meningitis surveillance in São Paulo [10].

In our analysis, limited to detection of HNS pathogens, PCR results were positive in 9% of HNS culture–negative CSF samples. Culture positivity was 3%, and concurrent culture positivity among HNS PCR–positive samples was 21%, emphasizing the value of PCR testing for bacterial meningitis surveillance in these countries. Inclusion of samples with C_t_ values of 36–39 increased the detection rate by 2%. The inclusion of these as laboratory-confirmed cases was based on (1) duplicate performance of PCR was with 2 positive results required to assign positivity and (2) a second, confirmatory PCR for serotyping/grouping, which assigned a serotype/group in 5% and 56% of these (high C_t_ value) *H. influenzae–* and *N. meningitidis*–positive samples, respectively, indicating that the HNS PCR results were not spurious.

Laboratory test methods are influenced by several factors, and no test is 100% sensitive or specific. CSF culture remains the reference standard for the diagnosis of bacterial meningitis; however, culture positivity has been shown to decline by 34% after the administration of antibiotics [25]. Both Mozambique and Brazil have documented an association between antibiotic use and negative culture results [10, 26]. Of culture-positive specimens, 75%– 90% of CSF are Gram stain positive, decreasing to 40%–60% in patients who have received antibiotics prior to lumbar puncture [27, 28]. The Gram stain is rapid and cheap, but clinical utility depends on bacterial load and the pathogen [29].

Rapid diagnostic tests differ in sensitivity and specificity, depending on the kit and pathogen of interest. BinaxNOW is highly sensitive for *S. pneumoniae* even after initiation of antibiotic treatment [30]; Pastorex, however, has been shown to be poor at detecting *S. pneumoniae* compared with culture and PCR, but with reasonable sensitivity and specificity for detecting *N. meningitidis* A and *N. meningitidis* W [31–33]. In contrast, PCR sensitivity for bacterial detection in CSF remains high for up to 1 week after antibiotic treatment [30]. In our analysis, an unusually high proportion of HNS PCR–positive samples were negative at routine microbiology testing, and this may be attributed to several factors, namely, specimen transport, laboratory capacity to perform the tests correctly and with suitable reagents, and/or timing of clinical presentation. All cases satisfied the clinical case definition of suspected bacterial meningitis.

Among HNS PCR–negative samples, 47 (0.8%) were reported as culture positive for either *H. influenzae, N. meningitidis,* or *S. pneumoniae*. These discrepancies could not be resolved, as culture was performed in-country, whereas PCR was performed in South Africa several months later. Sample mix-ups and laboratory processing errors (either at the primary diagnostic laboratory in-country or at the RRL) could not be definitively ruled out. *H. influenzae, N. meningitidis,* and *S. pneumoniae* are fastidious organisms that may not survive long transit times or variations in temperature, and refrigeration may prevent recovery of these organisms. Ideally, CSF specimens should be stored at room temperature or in an incubator (37ºC) if they cannot be processed immediately for culture.

Bacterial coinfection is rarely reported in meningitis cases [34]. Nevertheless, such diagnoses in individual patients should not be ignored but require careful investigation and correlation with clinical presentation. We cannot conclusively exclude laboratory or sample contamination as a possible reason for dual detection of pathogen targets in the 13 cases we reported in this analysis. At NICD, we have stringent control measures to minimize and detect contamination, which includes preparing an aliquot of CSF for molecular testing before performing routine phenotypic testing. In the current study, however, samples were processed for PCR after routine microbiology and chemistry testing performed in-country. Additional quality control measures included regular technical audits and provision of microbiology laboratory training (in-country).

At the NICD laboratory, RNP detection is used as a proxy for specimen quality and/or inhibition in clinical samples received for diagnosis or surveillance testing, and the absence of RNP in CSF is rare (<5%; data not shown) in South African samples. In this study, however, the percentage of RNP PCR negative samples was unusually (and consistently) high across all sites, for unclear reasons. CSF is hypotonic and therefore white blood cells are unstable and typically lyse within 2 hours [35]. Moreover, delays in sample processing in-country or prolonged storage (before shipment to South Africa) may have resulted in some degradation. Among samples that were HNS PCR negative, CSF turbidity and white blood cell count did not appear to differ vastly between those that were RNP positive and those that were RNP negative. As such, we think that the HNS PCR results for the RNP-negative CSF samples in this study were not likely false-negative for the targeted pathogens but rather had insufficient human cells for RNP to be detected by PCR. The global VP-IBD network reports all HNS PCR–negative samples as true-negatives, regardless of the RNP results [22].

This analysis has several limitations. Because not all CSF samples were sent for PCR, the data may not accurately reflect the true burden of meningitis caused by these 3 pathogens. Samples shipped were those that had complete data entries in the database and with adequate sample volume stored at the sentinel site. Second, PCR detection was limited to the 3 most common, vaccine-preventable meningitis-causing bacterial pathogens; other bacteria such as *Streptococcus agalactiae*, *Listeria monocytogenes,* and *Escherichia coli* commonly cause meningitis, especially in neonates [36, 37]. Multipathogen PCR detection platforms that simultaneously detect bacteria, viruses and fungi have the potential to revolutionize the diagnosis of common infectious syndromes. However, their utility in surveillance is currently hampered by low throughput and prohibitive cost [38, 39]. Some countries did not systematically collect or submit specimens annually, while low overall numbers of specimens were submitted from some countries.

Laboratory data for phenotypic testing were poorly captured or testing was not done for many samples, limiting comparisons to those samples for which laboratory data were available. We cannot rule out the possibility that we missed detecting a pathogen in some CSF samples, given that the specimens were received months after collection. There was no indication as to how long specimens took to reach the testing laboratory or whether they had been centrifuged, both of which could negatively influence pathogen detection by culture or PCR. This may also account, in part, for the absence or undetectable levels of RNP DNA in an unusually large proportion of CSF samples. We were not able to assess the impact of differences in storage temperature and storage duration on pathogen detection. To overcome some of these constraints, impregnating filter paper with CSF has been shown to be a useful alternative [40]. Finally, monitoring trends in antimicrobial resistance is an important aspect of surveillance but is currently reliant on bacterial cultures, and these data were not available for culture-negative samples. However, more advanced methods such as molecular detection of antibiotic resistance genes and next-generation sequencing of pathogens directly from clinical specimens are being used increasingly [41].

The Global IB-VPD Surveillance Network plays an important role in assisting laboratories to improve their microbiology capacity through regular on-site training and external quality assessment initiatives, in an effort to generate good-quality surveillance data. PCR-based detection was valuable in providing pathogen-specific and serotype/group data for suspected cases of vaccine-preventable bacterial meningitis in young children in African countries, where many challenges, such as lack of human and laboratory resources, impede pathogen detection and characterization.

## Supplementary Data

Supplementary materials are available at *The Journal of Infectious Diseases online*. Consisting of data provided by the authors to benefit the reader, the posted materials are not copyedited and are the sole responsibility of the authors, so questions or comments should be addressed to the corresponding author.

jiab092_suppl_Supplementary_Table_1

jiab092_suppl_Supplementary_Table_2

jiab092_suppl_Supplementary_Table_3

jiab092_suppl_Supplementary_Table_4

## Notes

***Acknowledgments.*** Thanks to Meseret Asefa Oda, Bacteriology National Reference Laboratory, Ethiopian Public Health Institute, Addis Ababa; Mwanaisha Seugendo, Bacteriology Laboratory, Bugando Medical Centre, Mwanza, United Republic of Tanzania; and William Mngube Patrick Mwengee and Ritha Willilo, World Health Organization Country Office, United Republic of Tanzania.

***Supplement sponsorship.*** This supplement is sponsored by the World Health Organization and the U. S. Centers for Disease Control and Prevention.

***Financial support.*** This work was supported by the World Health Organization and Gavi, the Vaccine Alliance (Data Strategic Focus Area grant)

***Potential conflicts of interest.*** All authors: No reported conflicts. All authors have submitted the ICMJE Form for Disclosure of Potential Conflicts of Interest. Conflicts that the editors consider relevant to the content of the manuscript have been disclosed.
