# Supplementary material for: The Role of Molecular Testing in Pediatric Meningitis Surveillance in Southern and East African Countries, 2008–2017
Source: J Infect Dis. 2021 Sep 1;224(Suppl 3):S194–203. doi: 10.1093/infdis/jiab092 (PMC8409535; doi:10.1093/infdis/jiab092)
Supplement: jiab092_suppl_Supplementary_Table_1 [file jiab092_suppl_supplementary_table_1.docx]

Supplementary Table 1. Real-time PCR reactions for serotyping of *Streptococcus pneumoniae*

| PCR reaction | Serotype/group detected |
| --- | --- |
| 1 | 1, 5, 23F |
| 2 | 4, 6A/6B/6C/6D, 9A/9V |
| 3 | 14, 18A/18B/18C/18F, 19F |
| 4 | 3, 7A/7F |
| 5 | 6C/6D, 12A/12B/12F/44/46, 22A/22F |
| 6 | 15A/15F, 23A, 33A/33F/37 |
| 7 | 2, 11A/11D, 16F |
| 8 | 8, 19A |
