# Supplementary material for: The Role of Molecular Testing in Pediatric Meningitis Surveillance in Southern and East African Countries, 2008–2017
Source: J Infect Dis. 2021 Sep 1;224(Suppl 3):S194–203. doi: 10.1093/infdis/jiab092 (PMC8409535; doi:10.1093/infdis/jiab092)
Supplement: jiab092_suppl_Supplementary_Table_2 [file jiab092_suppl_supplementary_table_2.docx]

Supplementary Table 2.

Country-level PCR positivity rates and serotype/group for bacterial meningitis-causing pathogens *S. pneumoniae*, *N. meningitidis* and *H. influenzae* in children aged less than 5 years, 2008-2017

| **Angola (N=2042)** | Sample collection year | | | | | | | | | | |
| --- | --- | --- | --- | --- | --- | --- | --- | --- | --- | --- | --- |
|  | 2010 | 2011 | 2012 | 2013 | Pre PCV | 2014 | 2015 | 2016 | 2017 | Post PCV | Total |
| No. CSFs received | 119 | 1 | 0 | 1 | 121 | 0 | 7 | 1195 | 719 | 1921 | 2042 |
| No. *lytA* positive | 21 (18%) | 0 | 0 | 0 | 21 (18%) | 0 | 2 (29%) | 45 (4%) | 23 (3%) | 71 (4%) | 92 (5%) |
| No. PCV13^a^ serotype^b^ (n/N) | 18/20 (90%) | NA | NA | NA | 18/20 (90%) | NA | 1/2 (50%) | 27/37 (73%) | 5/18 (28%) | 33/57 (58%) | 51/77 (66%) |
| No. *ctrA* positive | 6 (5%) | 0 | 0 | 0 | 6 (5%) | 0 | 0 | 14 (1%) | 6 (1%) | 20 (1%) | 26 (1%) |
| Serogroup (n) | B (1) C (4) W (1) | NA | NA | NA | B (1) C (4) W (1) | NA | NA | B (6) C (6) W (1) | B (3) C (1) Y (2) | B (9) C (7) W (1) Y (2) | B (10) C (11)  W (2) Y (2) |
| No. *hpd* positive^c^ | 8 (7%) | 0 | 0 | 0 | 8 (7%) | 0 | 0 | 19 (2%) | 17 (2%) | 36 (1.9%) | 44 (2%) |
| Serotype (n) | a (1) b (6) NT^d^ (1) | NA | NA | NA | a (1) b (6) NT (1) | NA | NA | a (7) b (7) NT (1) | a (3) b (11) NT (1) | a (10) b (18) NT (2) | a (11) b (24) NT (3) |
| No. HNS PCR negative | 71 (60%) | 1 (100%) | 0 | 0 | 72 (60%) | 0 | 4 (57%) | 683 (57%) | 351 (49%) | 1038 (54%) | 1110 (54%) |
| No. *RNase P* PCR negative | 13 (11%) | 0 | 0 | 1 (100%) | 14 (12%) | 0 | 1 (14%) | 425 (36%) | 309 (43%) | 734 (38%) | 748 (37%) |
| No. not tested | 0 | 0 | 0 | 0 | 0 | 0 | 0 | 9 | 13 | 22 (1%) | 22 (1%) |

^a^ PCV13 was introduced in June 2013 ^b^ *LytA*-positive samples with C_t_ values ≥35 were excluded from PCR serotyping ^c^ *H. influenza*e serotype b vaccine was introduced in 2006

^d^ NT – non-typeable

| **Ethiopia (N=911)** | Sample collection year | | | | | | | | | | | | |
| --- | --- | --- | --- | --- | --- | --- | --- | --- | --- | --- | --- | --- | --- |
|  | 2008 | 2009 | 2010 | 2011 | Pre PCV | 2012 | 2013 | 2014 | 2015 | 2016 | 2017 | Post PCV | Total |
| No. CSFs received | 7 | 5 | 2 | 13 | 27 | 118 | 139 | 122 | 130 | 260 | 115 | 884 | 911 |
| No. *lytA* positive | 2 (29%) | 0 | 0 | 1 | 3 (11%) | 12 (10%) | 15 (11%) | 11 (9%) | 7 (5%) | 25 (10%) | 2 (2%) | 72 (8%) | 75 (8%) |
| No. PCV10^a^ serotype^b^ (n/N) | 1/2 (50%) | NA | NA | 0 | 1/2 (50%) | 2/6 (33%) (33%) | 0/7 | 2/10 (20%) | 1/7 (14%) | 3/11 (27%) | 0 | 8/41 (20%) | 9/43 (21%) |
| No. *ctrA* positive | 0 | 2 (40%) | 0 | 0 | 2 (7%) | 8 (7%) | 5 (4%) | 10 (8%) | 7 (5%) | 5 (2%) | 0 | 35 (7%) | 37 (4%) |
| Serogroup (n) | NA | A (2) | NA | NA | A (2) | W (6) | A (2) B (1) W (1) | A (2) B (1)  C (1) W (3) | B (1) W(6) | A (3) W (2) | NA | A (7) B (3)  C (1) W (18) | A (9) B (3)  C (1) W (18) |
| No. *hpd* positive^c^ | 0 (0%) | 1 | 1 | 1 | 3 (11%) | 5 (4%) | 11 (8%) | 12 (10%) | 5 (4%) | 3 (1%) | 1 (1%) | 37 (4%) | 40 (4%) |
| Serotype (n) | NA | a (1) | b (1) | b (1) | a (1) b (2) | a (1) f (1) | b (4) | a (1) b (9) | b (3) | b (2) | b (1) | a (2) b (19)  f (1) | a (3) b (21)  f (1) |
| No. HNS PCR negative | 2 (29%) | 1 | 0 | 7 | 10 (41%) | 71 (60%) | 51 (37%) | 69 (59%) | 93 (72%) | 133 (77%) | 70 (61%) | 487 (55%) | 497 (55%) |
| No. *RNase P* PCR negative | 3 (43%) | 1 | 1 | 4 | 9 (34%) | 23 (19%) | 60 (43%) | 22 (18%) | 18 (14%) | 92 (15%) | 40 (35%) | 255 (29%) | 264 (29%) |
| No. not tested | 0 | 0 | 0 | 0 | 0 | 0 | 0 | 0 | 0 | 2 (0.8%) | 2 (1.7%) | 4 (0.5%) | 4 (0.4%) |

^a^ PCV10 was introduced in October 2011

^b^ *LytA*-positive samples with C_t_ values ≥35 were excluded from PCR serotyping

^c^ *H. influenza*e serotype b vaccine was introduced in 2006

| **Lesotho (N=201)** | Sample collection year | | | | | | | | | |
| --- | --- | --- | --- | --- | --- | --- | --- | --- | --- | --- |
|  | 2010 | 2011 | 2012 | 2013 | 2014 | 2015 | Pre PCV | 2016 | Post PCV | Total |
| No. CSFs received | 3 | 3 | 7 | 49 | 47 | 69 | 178 | 23 | 23 | 201 |
| No. *lytA* positive | 2 (67%) | 0 | 1 (14%) | 11 (22%) | 8 (17%) | 9 (7%) | 31 | 2 (9%) | 2 (9%) | 33 (16%) |
| No. PCV13^a^ serotype^b^ (n/N) | 0 | NA | 0 | 7/10 (70%) (70%) | 1/6 (17%) (17%) | 3/5 (60%) | 11/24 (46%) | 2/2 (100%) | 2/2 (100%) | 13/26 (50%) |
| No. *ctrA* positive | 0 | 1 (33%) | 0 | 2 (4%) | 0 | 2 (3%) | 5 (3%) | 0 | 0 | 5 (2.5%) |
| Serogroup (n) | NA | W (1) | NA | A (1) W (1) | NA | W (1) Y (1) | A (1) W (3) Y (1) | NA | NA | A (1) W (3) Y (1) |
| No. *hpd* positive^c^ | 0 | 0 | 0 | 1 (2%) | 2 (4%) | 3 (4%) | 6 (3%) | 2 (9%) | 2 (9%) | 8 (4%) |
| Serotype (n) | NA | NA | NA | b | NT^d^ (2) | b (1) NT (1) | b (2) NT (3) | b (2) | b (2) | b (4) NT (3) |
| No. HNS PCR negative | 1 | 0 | 2 (29%) | 32 (20%) | 30 (64%) | 48 (70%) | 113 (63%) | 19 (83%) | 19 (83%) | 132 (66%) |
| No. *RNase P* PCR negative | 0 | 2 (67%) | 4 (57%) | 4 (8%) | 7 (15%) | 10 (14%) | 27 (15%) | 0 (0%) | 0 (0%) | 27 (13%) |
| No. not tested | 0 | 0 | 0 | 0 | 0 | 0 | 0 | 0 | 0 | 0 |

^a^ PCV13 was introduced in July 2015

^b^ *LytA*-positive samples with C_t_ values ≥35 were excluded from PCR serotyping

^c^ *H. influenzae* serotype b vaccine was introduced in 2008

^d^ NT – non-typeable

| **Madagascar (N=2067)** | Sample collection year | | | | | | | | |
| --- | --- | --- | --- | --- | --- | --- | --- | --- | --- |
|  | 2012 | Pre PCV | 2013 | 2014 | 2015 | 2016 | 2017 | Post PCV | Total |
| No. CSFs received | 207 | 207 | 302 | 351 | 492 | 458 | 257 | 1860 | 2067 |
| No. *lytA* positive | 46 (22%) | 46 (22%) | 18 (6%) | 16 (5%) | 23 (5%) | 7 (2%) | 5 (2%) | 69 (4%) | 115 (6%) |
| No. PCV13^a^ serotype^b^ (n/N) | 7/28 (32%) | 7/28 (32%) | 4/14 (29%) | 5/15 (33%) | 2/18 (11%) | 1/7 (14%) | 0/5 (0%) | 12/59 (20%) | 19/87 (22%) |
| No. *ctrA* positive | 0 | 0 | 0 | 0 | 0 | 1 | 2 (0.8%) | 3 (0.2%) | 3 (0.1%) |
| Serogroup (n) | NA | NA | NA | NA | NA | W (1) | W (2) | W (3) | W (3) |
| No. *hpd* positive^c^ | 1 | 1 | 0 | 1 | 6 | 1 | 1 (0.4%) | 9 (0.5%) | 10 (0.5%) |
| Serotype (n) | NT^d^ | NT | NA | 0 | b (1) NT (1) | NT | 0 | b (1) NT (2) | b (1) NT (3) |
| No. HNS PCR negative | 90 (43%) | 90 (43%) | 107 (35%) | 253 (72%) | 398 (81%) | 244 (53%) | 142 (55%) | 1144 (62%) | 1234 |
| No. *RNase P* PCR negative | 71 (34%) | 71 (34%) | 178 (59%) | 81 (23%) | 65 (13%) | 205 (45%) | 102 (40%) | 631 (34%) | 701 |
| No. not tested | 0 | 0 | 0 | 0 | 0 | 0 | 5 (2%) | 5 (0.3%) | 5 (0.2%) |

^a^ PCV10 was introduced in November 2012

^b^ *LytA*-positive samples with C_t_ values ≥35 were excluded from PCR serotyping

^c^ *H. influenzae* serotype b vaccine was introduced in 2008

^d^ NT – non-typeable

| **Mozambique (N=285)** | Sample collection year | | | | | | |
| --- | --- | --- | --- | --- | --- | --- | --- |
|  | 2013 | Pre PCV | 2014 | 2015 | 2016 | Post PCV | Total |
| No. CSFs received | 91 | 91 | 60 | 27 | 107 | 194 | 285 |
| No. *lytA* positive | 27 (30%) | 27 (30%) | 10 (17%) | 3 (11%) | 6 (6%) | 19 (10%) | 46 (16%) |
| No. PCV10^a^ serotype^b^ (n/N) | 9/21 (43%) | 9/21 (43%) | 4/9 (44%) | 0/3 (0%) | 1/5 (20%) | 5/17 (29%) | 14/38 (37%) |
| No. *ctrA* positive | 0 | 0 | 8 (13%) | 1 (4%) | 1 | 10 (11%) | 10 (3.5%) |
| Serogroup (n) | NA | NA | W (7) Y (1) | W | B | B (1) W (8) Y (1) | B (1) W (8) Y (1) |
| No. *hpd* positive^c^ | 3 (3%) | 3 (3%) | 1 (2%) | 0 | 2 (2%) | 3 (1.5) | 6 (2%) |
| Serotype (n) | b (1) | b (1) | a | NA | a (1) b (1) | a (2) b (1) | a (2) b (2) |
| No. HNS PCR negative | 50 (56%) | 50 (56%) | 33 (55%) | 15 (56%) | 65 (33%) | 113 (58%) | 163 (57%) |
| No. *RNase P* PCR negative | 11 (12%) | 11 (12%) | 8 (13%) | 8 (30%) | 33 (31%) | 49 (25%) | 60 (21%) |
| No. not tested | 0 | 0 | 0 | 0 | 0 | 0 | 0 |

^a^ PCV10 was introduced in April 2013

^b^ *LytA*-positive samples with C_t_ values ≥35 were excluded from PCR serotyping

^c^ *H. influenzae* serotype b vaccine was introduced in 2009

| **Namibia (N=683)** | Sample collection year | | | | | | | |
| --- | --- | --- | --- | --- | --- | --- | --- | --- |
|  | 2013 | 2014 | Pre PCV | 2015 | 2016 | 2017 | Post PCV | Total |
| No. CSFs received | 2 | 114 | 116 | 205 | 286 | 76 | 567 | 683 |
| No. *lytA* positive | 0 | 12 (10%) | 12 (10%) | 6 (3%) | 2 (1%) | 2 (3%) | 10 (2%) | 22 |
| No. PCV13^a^ serotype^b^ (n/N) | NA | 4/9 (44%) | 4/9 (44%) | 2/3 (67%) | 2/2 (100%) | 0/0 (0%) | 4/5 (80%) | 8/14 (57%) |
| No. *ctrA* positive | 0 | 0 | 0 | 0 | 0 | 0 | 0 | 0 |
| Serogroup (n) | NA | NA | NA | NA | NA | NA | NA | NA |
| No. *hpd* positive^c^ | 0 | 6 (5%) | 6 (5%) | 2 (1%) | 4 (1%) | 1 (1%) | 7 (1.2%) | 13 |
| Serotype (n) | NA | b (2) | b (2) | 0 | b (4) | 0 | b (4) | b (6) |
| No. HNS PCR negative | 1 | 54 (47%) | 55 (47%) | 144 (70%) | 171 (60%) | 37 (49%) | 352 62%) | 407 |
| No. *RNase P* PCR negative | 1 | 42 (37%) | 43 (36%) | 53 (26%) | 109 (38%) | 36 (47%) | 198 (35%) | 241 |
| No. not tested | 0 | 0 | 0 | 0 | 0 | 0 | 0 | 0 |

^a^ PCV13 was introduced in November 2014

^b^ *LytA*-positive samples with C_t_ values ≥35 were excluded from PCR serotyping

^c^ *H. influenzae* serotype b vaccine was introduced in 2009

| **Rwanda** **(N=97)** | Sample collection year | | | |
| --- | --- | --- | --- | --- |
|  | 2015 | 2016 | 2017 | Total (Post PCV) |
| No. samples receive | 47 | 41 | 9 | 97 |
| No. *lytA* positive | 0 | 2 | 0 | 2 (2%) |
| No. PCV13^a^ serotype (n/N) | NA | 1/2 (50%) | NA | 1/2 (50%) |
| No. *ctrA* positive | 3 (6%) | 1 (2%) | 0 (0%) | 4 (4%) |
| Serogroup (n) | W (3) | W | NA | W (4) |
| No. *hpd* positive^c^ | 0 (0%) | 1 (2%) | 0 (0%) | 1 (1%) |
| Serotype (n) | NA | 0 | NA | 0 |
| No. PCR negative | 30 (63%) | 34 (83%) | 8 (89%) | 72 (74%) |
| No. *RNase P* PCR negative | 14 (29%) | 3 (7%) | 1 (11%) | 18 (19%) |
| No. not tested | 0 | 0 | 0 | 0 |

^a^ PCV7 was introduced in July 2009 and replaced with PCV13 in August 2011

^b^ *LytA*-positive samples with C_t_ values ≥35 were excluded from PCR serotyping

^c^ *H. influenzae* serotype b vaccine was introduced in 2002

| **eSwatini (Swaziland)** **(N=197)** | Sample collection year | | | | | | | | |
| --- | --- | --- | --- | --- | --- | --- | --- | --- | --- |
|  | 2012 | 2013 | 2014 | Pre PCV | 2015 | 2016 | 2017 | Post PCV | Total |
| No. samples received | 19 | 26 | 42 | 87 | 40 | 31 | 39 | 110 | 197 |
| No. *lytA* positive | 1 (5%) | 3 (10%) | 1 (2%) | 5 (6%) | 0 (0%) | 1 (3%) | 1 (3%) | 2 (2%) | 7 (4%) |
| No. PCV13^a^ serotype^b^ (n/N) | 1/1 (100%) | 1/2 (50%) | 0/1 (0%) | 2/4 (50%) | NA | 0/1 | 0/1 | 0/2 | 2/6 (33%) |
| No. *ctrA* positive | 0 (0%) | 0 (0%) | 0 (0%) | 0 (0%) | 0 (0%) | 0 (0%) | 2 (5%) | 2 (2%) | 2 (1%) |
| Serogroup (n) | NA | NA | NA | NA | NA | NA | Y (1) W (1) | Y (1) W (1) | Y (1) W (1) |
| No. *hpd* positive^c^ | 1 (5%) | 0 (0%) | 2 (5%) | 3 | 1 (3%) | 1 (3%) | 1 (3%) | 3 | 6 (3%) |
| Serotype^b^ (n) | b | NA | a (1) | a (1) b (1) | b | b | 0 | b (2) | a (1) b (3) |
| No. PCR negative | 1 (5%) | 8 (31%) | 19 (45%) | 28 (32%) | 18 (45%) | 13 (42%) | 10 (26%) | 41 (37%) | 69 (35%) |
| No. *RNase P* PCR negative | 16 (84%) | 15 (58%) | 20 (48%) | 51 (59%) | 21 (53%) | 16 (52%) | 24 (62%) | 61 (55%) | 112 (57%) |
| No. not tested | 0 | 0 | 0 | 0 | 0 | 0 | 1 | 1 | 1 |

^a^ PCV13 was introduced in April 2014

^b^ *LytA*-positive samples with C_t_ values ≥35 were excluded from PCR serotyping

^c^ *H. influenzae* serotype b vaccine was introduced in 2009

| **Tanzania (N=51)** | Sample collection year | | | | | |
| --- | --- | --- | --- | --- | --- | --- |
|  | 2014 | 2015 | | | 2016 | Total (Post PCV) |
| No. samples | 17 | | 29 | 5 | | 51 |
| No. *lytA* positive | 0 | | 1 (3%) | 1 (20%) | | 2 (4%) |
| No. PCV13^a^ serotype (n/N) | NA | | 1/1 (100%) | 0 | | 1/2 (50%) |
| No. *ctrA* positive | 0 | | 0 | 1 (20%) | | 1 (2%) |
| Serogroup (n) | NA | | NA | B | | B (1) |
| No. *hpd* positive^b^ | 0 | | 0 | 0 | | 0 |
| Serotype (n) | NA | | NA | NA | | NA |
| No. PCR negative | 12 (71%) | | 17 (58%) | 3 (60%) | | 32 (63%) |
| No. *RNase P* PCR negative | 5 (29%) | | 11 (38%) | 0 | | 16 (31%) |
| No. not tested | 0 | | 0 | 0 | | 0 |

^a^ PCV13 was introduced in December 2012

^b^ *H. influenzae* serotype b vaccine was introduced in 2009

| **Uganda** **(N=2998)** | Sample collection year | | | | | | | | |
| --- | --- | --- | --- | --- | --- | --- | --- | --- | --- |
|  | 2012 | 2013 | Pre PCV | 2014 | 2015 | 2016 | 2017 | Post PCV | Total |
| No. samples received | 542 | 810 | 1352 | 491 | 600 | 297 | 258 | 1646 | 2998 |
| No. *lytA* positive | 73 (15%) | 85 (11%) | 158 (12%) | 24 (5%) | 36 (6%) | 22 (7%) | 14 (5%) | 96 (6%) | 254 (8%) |
| No. PCV10^a^ serotype^b^ (n/N) | 35/54 (65%) | 44/57 (76%) | 79/111 (71%) | 9/19 (47%) | 16/32 (50%) | 6/20 (30%) | 4/11 (36%) | 35/82 (43%) | 114/193 (59%) |
| No. *ctrA* positive | 0 | 13 (2%) | 13 (1%) | 12 (2%) | 12 (2%) | 1 (0.3%) | 7 (3%) | 32 (2%) | 45 (1.5%) |
| Serogroup (n) | NA | W (12) | W (12) | W (12) | W (10) | 0 | C (1) W (4)  Y (2) | C (1) W (26)  Y (2) | C (1) W (38)  Y (2) |
| No. *hpd* positive^c^ | 21 (4%) | 22 (3%) | 43 (3%) | 13 (3%) | 12 (2%) | 10 (3%) | 4 (1.5%) | 39 (2%) | 82 |
| Serotype (n) | a (2) b (11) c (1) | a (3) b (9) c (1) | a (5) b (20)  c (2) | a (2) b (4) | a (2) b (6) NT^d^ (1) | a (1) b (7) c (1) | b (4) | a (5) b (21)  c (1) NT (1) | a (10) b (41)  c (3) NT (1) |
| No. PCR negative | 253(46%) | 445 (55%) | 698 (52%) | 308 (63%) | 408 (68%) | 179 (60%) | 148 (57%) | 1043 (63%) | 1741 (58%) |
| No. inconclusive | 196 (36%) | 247 (30%) | 443 (33%) | 135 (27%) | 132 (22%) | 85 (29%) | 85 (33%) | 437 (27%) | 880 (3%) |
| No. not tested | 0 | 0 | 0 | 0 | 0 | 0 | 0 | 0 | 0 |

^a^ PCV10 was introduced in April 2013

^b^ *LytA*-positive samples with C_t_ values ≥35 were excluded from PCR serotyping

^c^ *H. influenzae* serotype b vaccine was introduced in 2002

^d^ NT – nontypeable

| **Zambia** **(N=1148)** |  |  | | Sample collection year | | | | | |  |  |
| --- | --- | --- | --- | --- | --- | --- | --- | --- | --- | --- | --- |
|  | 2011 | 2012 | 2013 | | Pre PCV | 2014 | 2015 | 2016 | 2017 | Post PCV | Total |
| No. samples received | 1 | 181 | 199 | | 381 | 222 | 188 | 226 | 132 | 767 | 1148 |
| No. *lytA* positive | 1 | 19 (11%) | 27 (14%) | | 37 (10%) | 19 (9%) | 11 (6%) | 11 (5%) | 8 (6%) | 49 (6%) | 96 (8%) |
| No. PCV10^a^ serotype^b^ (n/N) | 1/1 (100%) | 9/16 (56%) | 13/23 (57%) | | 23/40 (58%) | 9/16 (56%) | 6/11 (55%) | 5/9 (56%) | 3/8 (38%) | 23/44 (52%) | 46/84 (55%) |
| No. *ctrA* positive | 0 | 2 (2%) | 9 (5%) | | 11 (3%) | 12 (5%) | 11 (6%) | 3 (1%) | 1 (1%) | 27 (4%) | 38 (3%) |
| Serogroup (n) | NA | W (2) | W (9) | | W (11) | W (12) | W (11) | W (2) | W (1) | W (26) | W (37) |
| No. *hpd* positive^c^ | 0 | 3 (1%) | 5 (3%) | | 8 (2%) | 9 (4%) | 7 (4%) | 5 (2%) | 1 (1%) | 22 (3%) | 30 (3%) |
| Serotype^c^ (n) | NA | b (3) | b (4) | | b (7) | b (5) | a (1) b (5) | b (4) | a (1) | a (2) b (14) | a (2) b (21) |
| No. PCR negative | 0 | 104 (57%) | 100 (50%) | | 203 (53%) | 133 (60%) | 130 (69%) | 154 (68%) | 73 (55%) | 490 (64%) | 693 (60%) |
| No. *RNase P* PCR negative | 0 | 54 (30%) | 59 (30%) | | 113 (30%) | 48 (22%) | 29 (15%) | 53 (23%) | 49 (37%) | 179 (23%) | 292 (25%) |
| No. not tested | 0 | 0 | 0 | | 0 | 0 | 0 | 0 | 0 | 0 | 0 |

^a^ PCV10 was introduced in May 2013

^b^ *LytA*-positive samples with C_t_ values ≥35 were excluded from PCR serotyping

^c^ *H. influenzae* serotype b vaccine was introduced in 2004

| **Zimbabwe (N=1000)** | Sample collection year | | | | | | | | | |
| --- | --- | --- | --- | --- | --- | --- | --- | --- | --- | --- |
|  | 2011 | 2012 | Pre PCV | 2013 | 2014 | 2015 | 2016 | 2017 | Post PCV | Total |
| No. samples received | 37 | 239 | 276 | 64 | 97 | 219 | 185 | 159 | 724 | 1000 |
| No. *lytA* positive | 7 (19%) | 17 (7%) | 24 (9%) | 7 (11%) | 4 (4%) | 13 (6%) | 3 (2%) | 2 (1%) | 29 (4%) | 53 (5%) |
| No. PCV13^a^ serotype^b^ (n/N) | 3/3 (100%) | 9/14 (64%) | 12/17 (71%) | 3/6 (50%) | 1/2 (50%) | 4/9 (44%) | 0/3 (0%) | 0/2 (0%) | 8/22 (36%) | 20/39 (51%) |
| No. *ctrA* positive | 1 (3%) | 1 (0.4%) | 2 (0.7%) | 0 | 2 (2%) | 0 | 0 | 1 (0.6%) | 3 (0.4%) | 5 (0.5%) |
| Serogroup (n) | W | W | W (2) | NA | B (1) W (1) | NA | NA | W | B (1) W (2) | B (1) W (4) |
| No. *hpd* positive^c^ | 0 | 7 (3%) | 7 (3%) | 0 | 1 (1%) | 1 (0.5%) | 1 (0.5%) | 1 (0.6%) | 4 | 11 |
| Serotype^b^ (n) | NA | a (1) b (4) | a (1) b (4) | NA | 0 | 0 | 0 | b | b (1) | a (1) b (5) |
| No. PCR negative | 13 (34%) | 91 (37%) | 102 (37%) | 17 (26%) | 40 (40%) | 109 (50%) | 59 (32%) | 63 (40%) | 288 (40%) | 390 (39%) |
| No. *RNase P* PCR negative | 20 (53%) | 123 (49%) | 141 (51%) | 40 (62%) | 50 (50%) | 96 (44%) | 122 (66%) | 83 (52%) | 391 (54%) | 532 (53%) |
| No. not tested (tube empty) | 0 | 0 | 0 | 0 | 0 | 0 | 0 | 9 | 0 | 0 |

^a^ PCV13 was introduced in July 2012

^b^ *LytA*-positive samples with C_t_ values ≥35 were excluded from PCR serotyping

^c^ *H. influenzae* serotype b vaccine was introduced in 2008
