# Supplementary material for: The Role of Molecular Testing in Pediatric Meningitis Surveillance in Southern and East African Countries, 2008–2017
Source: J Infect Dis. 2021 Sep 1;224(Suppl 3):S194–203. doi: 10.1093/infdis/jiab092 (PMC8409535; doi:10.1093/infdis/jiab092)
Supplement: jiab092_suppl_Supplementary_Table_3 [file jiab092_suppl_supplementary_table_3.docx]

Supplementary Table 3. Summary of PCR results for 13 CSF samples with dual-target detections for *H. influenzae, N. meningitidis* and/or *S. pneumoniae*

| Sample ID | *hpd* C_t_^1^ value  (*H. influenzae*) | *H. influenzae* serotype | *ctrA* C_t_^1^ value  (*N. meningitidis*) | *N. meningitidis* serogroup | *lytA* C_t_^1^ value  (*S. pneumoniae*) | *S. pneumoniae* serotype | Final PCR result |
| --- | --- | --- | --- | --- | --- | --- | --- |
| 1321 | 35 | b | - | - | 22 | Neg38 | *hpd + lytA* |
| 3034 | 37 | Not assigned^2^ | - | - | 38 | Not done^3^ | *hpd + lytA* |
| 3024 | 37 | Not assigned^2^ | - | - | 37 | Not done^3^ | *hpd + lytA* |
| 3764 | 39 | Not assigned^2^ | - | - | 39 | Not done^3^ | *hpd + lytA* |
| 1684 | 28 | b | 18 | A | - | - | *hpd + ctrA* |
| 3981 | - | - | 23 | W | 22 | 6A/6B | *ctrA + lytA* |
| 543 | 35 | b | - | - | 34 | Neg38 | *hpd + lytA* |
| 3972 | - | - | 31 | W | 29 | 6A/6B | *ctrA + lytA* |
| 3782 | - | - | 31 | C | 30 | 3 | *ctrA + lytA* |
| 2986 | 12 | b | - | - | 15 | 3 | *hpd + lytA* |
| 57 | 32 | Non-typeable | - | - | 33 | 23F | *hpd + lytA* |
| 3923 | - | - | 30 | W | 19 | Neg38 | *ctrA + lytA* |
| 6489 | 33 | b | 35 | B | - | - | *hpd + ctrA* |

^1^ C_t_ – PCR cycle threshold

^2^ Not assigned, presumably due to low bacterial load (*hpd* C_t_ value >35)

^3^ *S. pneumoniae* serotyping was only conducted on *lytA*-positive samples with C_t_ values ≤35
