# Supplementary material for: The Role of Molecular Testing in Pediatric Meningitis Surveillance in Southern and East African Countries, 2008–2017
Source: J Infect Dis. 2021 Sep 1;224(Suppl 3):S194–203. doi: 10.1093/infdis/jiab092 (PMC8409535; doi:10.1093/infdis/jiab092)
Supplement: jiab092_suppl_Supplementary_Table_4 [file jiab092_suppl_supplementary_table_4.docx]

**Supplementary Table 4A**. CSF characteristics associated with HNS PCR positive results in children <5 years, Invasive Bacterial Vaccine-Preventable Diseases (IB-VPD) surveillance, southern and east Africa, 2008-2017

| Characteristic |  | HNS^a^ PCR positive | | | HNS^a^ PCR negative | | | Adjusted  odds ratio^b^  (95% CI) | P value | |
| --- | --- | --- | --- | --- | --- | --- | --- | --- | --- | --- |
|  | | Total^c^  N=1195  n (%)^c^ | C_t_≤35 (N=910)  n (%)^c^ | C_t_ 36-39  (N=285)  n (%)^c^ | Total^c^  N=10,299  n (%)^c^ | RNase P PCR C_t_ ≤35 (N=6405)  n (%)^c^ | RNase P PCR C_t_ ≥36 (N=3894)  n (%)^c^ |  | |  |
| CSF appearance | |  |  |  |  |  |  |  | |  |
| Clear | | 437 (52) | 277 (43) | 160 (79) | 5319 (76) | 3128 (72) | 2191 (84) | Ref | | - |
| Turbid | | 275 (33) | 258 (40) | 17 (8) | 514 (7) | 400 (9) | 114 (4) | **6.29 (5.09-7.76)** | | **<0.001** |
| Bloody | | 63 (8) | 55 (9) | 8 (4) | 613 (9) | 440 (10) | 173 (7) | 1.08 (0.78-1.49) | | 0.656 |
| Xanthochromic | | 65 (8) | 48 (8) | 17 (8) | 527 (8) | 382 (9) | 145 (6) | **1.54 (1.13-2.11)** | | **0.007** |
| Unknown | | 355 (30) | 272 (30) | 83 (29) | 3326 (32) | 2055 (32) | 1271 (33) | - | | - |
| White blood cell count (cells/mm^3^) | |  |  |  |  |  |  |  | |  |
| <10 | | 477 (53) | 298 (44) | 179 (81) | 6415 (84) | 3756 (82) | 2659 (90) | Ref | | - |
| 10-100 | | 317 (35) | 284 (42) | 33 (15) | 908 (12) | 674 (15) | 234 (8) | **4.57 (3.77-5.54)** | | **<0.001** |
| >100 | | 103 (11) | 95 (14) | 8 (4) | 212 (3) | 164 (4) | 48 (2) | **5.92 (4.40-7.97)** | | **<0.001** |
| Unknown | | 298 (25) | 233 (26) | 65 (23) | 2764 (27) | 1811 (28) | 953 (24) | - | | - |
| Protein (mg/dL) | |  |  |  |  |  |  |  | |  |
| ≤100 | | 254 (70) | 190 (65) | 64 (88) | 3768 (91) | 2294 (90) | 1474 (92) | Ref | | - |
| >100 | | 110 (30) | 101 (35) | 9 (12) | 383 (9) | 257 (10) | 126 (8) | **6.66 (4.80-9.25)** | | **<0.001** |
| Unknown | | 831 (70) | 619 (68) | 212 (74) | 6148 (60) | 3854 (60) | 2294 (59) | - | | - |
| Culture | |  |  |  |  |  |  |  | |  |
| Hi, Sp or Nm^d^ | | 160 (22) | 159 (29) | 1 (0.5) | 43 (0.7) | 26 (1) | 17 (0.7) | **32.28 (20.69-50.34)** | | **<0.001** |
| Other bacteria | | 20 (3) | 15 (3) | 5 (3) | 124 (2) | 76 (2) | 48 (2) | 1.26 (0.70-2.28) | | 0.446 |
| Negative | | 558 (76) | 375 (68) | 183 (97) | 5759 (97) | 3496 (97) | 2263 (97) | Ref | | - |
| Unknown | | 457 (38) | 361 (40) | 96 (34) | 4373 (42) | 2807 (44) | 1566 (40) | - | | - |
| Rapid diagnostic test | |  |  |  |  |  |  |  | |  |
| Hi, Sp or Nm^b^ | | 136 (67) | 128 (78) | 8 (22) | 504 (24) | 321 (25) | 183 (21) | **16.45 (9.81-27.59)** | | **<0.001** |
| Other bacteria | | 1 (0.5) | 1 (0.6) | 0 (0) | 14 (0.7) | 11 (1) | 3 (0.3) | 1.33 (0.13-13.92) | | 0.810 |
| Negative | | 65 (32) | 36 (22) | 29 (78) | 1626 (76) | 939 (74) | 687 (79) | Ref | | - |
| Unknown | | 993 (83) | 745 (82) | 248 (87) | 8155 (79) | 5134 (80) | 3021 (78) | - | | - |
| Gram stain | |  |  |  |  |  |  |  | |  |
| Gram-pos cocci | | 154 (26) | 151 (34) | 3 (2) | 186 (4) | 116 (5) | 70 (4) | **9.14 (6.85-12.20)** | | **<0.001** |
| Gram-neg bacilli/coccobacilli | | 73 (12) | 53 (12) | 20 (13) | 472 (11) | 252 (10) | 220 (12) | 1.39 (0.98-1.99) | | 0.068 |
| Gram-neg cocci | | 23 (4) | 21 (5) | 2 (1) | 77 (2) | 25 (1) | 52 (3) | **6.28 (3.27-12.03)** | | **<0.001** |
| Neg/Nothing of significance | | 341 (58) | 212 (48) | 129 (84) | 3604 (83) | 2166 (84) | 1438 (81) | Ref | | - |
| Unknown | | 604 (51) | 473 (52) | 131 (46) | 5960 (58) | 3846 (60) | 2114 (54) | - | | - |

^a^ HNS = multiplex PCR which detects *H. influenzae*, *N. meningitidis* and *S. pneumoniae*

^b^ Adjusted odds ratio, controlling for geographic location (country), comparing characteristics by HNS results (HNS positive vs HNS negative), including only samples with RNase P C_t_<36

^c^ Denominators used to calculate % for each characteristic excluded those samples with data unknown

^d^ Hi – *H. influenzae*, Sp – *S. pneumoniae*, Nm – *N. meningitidis*

**Supplementary Table 4B**. CSF characteristics associated with HNS PCR positive results in children <5 years, Invasive Bacterial Vaccine-Preventable Diseases (IB-VPD) surveillance, southern and east Africa, 2008-2017

| Characteristic |  | HNS^a^ PCR positive | | | HNS^a^ PCR negative | | | Adjusted  odds ratio^b^  (95% CI) | P value | |
| --- | --- | --- | --- | --- | --- | --- | --- | --- | --- | --- |
|  | | Total^c^  N=1195  n (%)^c^ | C_t_≤35 (N=910)  n (%)^c^ | C_t_ 36-39  (N=285)  n (%)^c^ | Total^c^  N=10,299  n (%)^c^ | RNase P PCR C_t_ ≤35 (N=6405)  n (%)^c^ | RNase P PCR C_t_ ≥36 (N=3894)  n (%)^c^ |  | |  |
| CSF appearance | |  |  |  |  |  |  |  | |  |
| Clear | | 437 (52) | 277 (43) | 160 (79) | 5319 (76) | 3128 (72) | 2191 (84) | Ref | | - |
| Turbid | | 275 (33) | 258 (40) | 17 (8) | 514 (7) | 400 (9) | 114 (4) | **8.49 (4.95-14.58)** | | **<0.001** |
| Bloody | | 63 (8) | 55 (9) | 8 (4) | 613 (9) | 440 (10) | 173 (7) | **3.86 (1.77-8.41)** | | **0.001** |
| Xanthochromic | | 65 (8) | 48 (8) | 17 (8) | 527 (8) | 382 (9) | 145 (6) | 1.32 (0.71-2.46) | | 0.376 |
| Unknown | | 355 (30) | 272 (30) | 83 (29) | 3326 (32) | 2055 (32) | 1271 (33) | - | | - |
| White blood cell count (cells/mm^3^) | |  |  |  |  |  |  |  | |  |
| <10 | | 477 (53) | 298 (44) | 179 (81) | 6415 (84) | 3756 (82) | 2659 (90) | Ref | | - |
| 10-100 | | 317 (35) | 284 (42) | 33 (15) | 908 (12) | 674 (15) | 234 (8) | **5.23 (3.40-8.04)** | | **<0.001** |
| >100 | | 103 (11) | 95 (14) | 8 (4) | 212 (3) | 164 (4) | 48 (2) | **6.97 (3.28-14.83)** | | **<0.001** |
| Unknown | | 298 (25) | 233 (26) | 65 (23) | 2764 (27) | 1811 (28) | 953 (24) | - | | - |
| Protein (mg/dL) | |  |  |  |  |  |  |  | |  |
| ≤100 | | 254 (70) | 190 (65) | 64 (88) | 3768 (91) | 2294 (90) | 1474 (92) | Ref | | - |
| >100 | | 110 (30) | 101 (35) | 9 (12) | 383 (9) | 257 (10) | 126 (8) | **3.84 (1.66-8.85)** | | **0.002** |
| Unknown | | 831 (70) | 619 (68) | 212 (74) | 6148 (60) | 3854 (60) | 2294 (59) | - | | - |
| Culture | |  |  |  |  |  |  |  | |  |
| Hi, Sp or Nm^d^ | | 160 (22) | 159 (29) | 1 (0.5) | 43 (0.7) | 26 (1) | 17 (0.7) | **69.21 (9.58-500.17)** | | **<0.001** |
| Other bacteria | | 20 (3) | 15 (3) | 5 (3) | 124 (2) | 76 (2) | 48 (2) | 0.70 (0.22-2.19) | | 0.538 |
| Negative | | 558 (76) | 375 (68) | 183 (97) | 5759 (97) | 3496 (97) | 2263 (97) | Ref | | - |
| Unknown | | 457 (38) | 361 (40) | 96 (34) | 4373 (42) | 2807 (44) | 1566 (40) | - | | - |
| Rapid diagnostic test | |  |  |  |  |  |  |  | |  |
| Hi, Sp or Nm^b^ | | 136 (67) | 128 (78) | 8 (22) | 504 (24) | 321 (25) | 183 (21) | **17.01 (6.39-45.30)** | | **<0.001** |
| Other bacteria | | 1 (0.5) | 1 (0.6) | 0 (0) | 14 (0.7) | 11 (1) | 3 (0.3) | - | | - |
| Negative | | 65 (32) | 36 (22) | 29 (78) | 1626 (76) | 939 (74) | 687 (79) | Ref | | - |
| Unknown | | 993 (83) | 745 (82) | 248 (87) | 8155 (79) | 5134 (80) | 3021 (78) | - | | - |
| Gram stain | |  |  |  |  |  |  |  | |  |
| Gram-pos cocci | | 154 (26) | 151 (34) | 3 (2) | 186 (4) | 116 (5) | 70 (4) | **29.67 (9.16-96.11)** | | **<0.001** |
| Gram-neg bacilli/coccobacilli | | 73 (12) | 53 (12) | 20 (13) | 472 (11) | 252 (10) | 220 (12) | 1.05 (0.56-1.99) | | 0.875 |
| Gram-neg cocci | | 23 (4) | 21 (5) | 2 (1) | 77 (2) | 25 (1) | 52 (3) | **5.06 (1.14-22.46)** | | **0.033** |
| Neg/Nothing of significance | | 341 (58) | 212 (48) | 129 (84) | 3604 (83) | 2166 (84) | 1438 (81) | Ref | | - |
| Unknown | | 604 (51) | 473 (52) | 131 (46) | 5960 (58) | 3846 (60) | 2114 (54) | - | | - |

^a^ HNS = multiplex PCR which detects *H. influenzae*, *N. meningitidis* and *S. pneumoniae*

^b^ Adjusted odds ratio, controlling for geographic location (country), comparing characteristics by HNS results (HNS positive C_t_<36 vs C_t_>36)

^c^ Denominators used to calculate % for each characteristic excluded those samples with data unknown

^d^ Hi – *H. influenzae*, Sp – *S. pneumoniae*, Nm – *N. meningitidis*

**Supplementary Table 4C**. CSF characteristics associated with RNase P PCR results in children <5 years, Invasive Bacterial Vaccine-Preventable Diseases (IB-VPD) surveillance, southern and east Africa, 2008-2017

| Characteristic |  | HNS^a^ PCR positive | | | HNS^a^ PCR negative | | | Adjusted  odds ratio^b^  (95% CI) | P value | |
| --- | --- | --- | --- | --- | --- | --- | --- | --- | --- | --- |
|  | | Total^c^  N=1195  n (%)^c^ | C_t_≤35 (N=910)  n (%)^c^ | C_t_ 36-39  (N=285)  n (%)^c^ | Total^c^  N=10,299  n (%)^c^ | RNase P PCR C_t_ ≤35 (N=6405)  n (%)^c^ | RNase P PCR C_t_ ≥36 (N=3894)  n (%)^c^ |  | |  |
| CSF appearance | |  |  |  |  |  |  |  | |  |
| Clear | | 437 (52) | 277 (43) | 160 (79) | 5319 (76) | 3128 (72) | 2191 (84) | Ref | | - |
| Turbid | | 275 (33) | 258 (40) | 17 (8) | 514 (7) | 400 (9) | 114 (4) | **2.27 (1.82-2.82)** | | **<0.001** |
| Bloody | | 63 (8) | 55 (9) | 8 (4) | 613 (9) | 440 (10) | 173 (7) | **1.93 (1.59-2.33)** | | **<0.001** |
| Xanthochromic | | 65 (8) | 48 (8) | 17 (8) | 527 (8) | 382 (9) | 145 (6) | **1.95 (1.59-2.40)** | | **<0.001** |
| Unknown | | 355 (30) | 272 (30) | 83 (29) | 3326 (32) | 2055 (32) | 1271 (33) | - | | - |
| White blood cell count (cells/mm^3^) | |  |  |  |  |  |  |  | |  |
| <10 | | 477 (53) | 298 (44) | 179 (81) | 6415 (84) | 3756 (82) | 2659 (90) | Ref | | - |
| 10-100 | | 317 (35) | 284 (42) | 33 (15) | 908 (12) | 674 (15) | 234 (8) | **2.19 (1.86-2.57)** | | **<0.001** |
| >100 | | 103 (11) | 95 (14) | 8 (4) | 212 (3) | 164 (4) | 48 (2) | **2.15 (1.54-2.99)** | | **<0.001** |
| Unknown | | 298 (25) | 233 (26) | 65 (23) | 2764 (27) | 1811 (28) | 953 (24) | - | | - |
| Protein (mg/dL) | |  |  |  |  |  |  |  | |  |
| ≤100 | | 254 (70) | 190 (65) | 64 (88) | 3768 (91) | 2294 (90) | 1474 (92) | Ref | | - |
| >100 | | 110 (30) | 101 (35) | 9 (12) | 383 (9) | 257 (10) | 126 (8) | **1.26 (1.00-1.60)** | | **0.049** |
| Unknown | | 831 (70) | 619 (68) | 212 (74) | 6148 (60) | 3854 (60) | 2294 (59) | - | | - |
| Culture | |  |  |  |  |  |  |  | |  |
| Hi, Sp or Nm^d^ | | 160 (22) | 159 (29) | 1 (0.5) | 43 (0.7) | 26 (1) | 17 (0.7) | 0.95 (0.51-1.78) | | 0.874 |
| Other bacteria | | 20 (3) | 15 (3) | 5 (3) | 124 (2) | 76 (2) | 48 (2) | 0.98 (0.66-1.44) | | 0.907 |
| Negative | | 558 (76) | 375 (68) | 183 (97) | 5759 (97) | 3496 (97) | 2263 (97) | Ref | | - |
| Unknown | | 457 (38) | 361 (40) | 96 (34) | 4373 (42) | 2807 (44) | 1566 (40) | - | | - |
| Rapid diagnostic test | |  |  |  |  |  |  |  | |  |
| Hi, Sp or Nm^b^ | | 136 (67) | 128 (78) | 8 (22) | 504 (24) | 321 (25) | 183 (21) | **1.57 (1.10-2.23)** | | **0.012** |
| Other bacteria | | 1 (0.5) | 1 (0.6) | 0 (0) | 14 (0.7) | 11 (1) | 3 (0.3) | 2.86 (0.72-11.27) | | 0.134 |
| Negative | | 65 (32) | 36 (22) | 29 (78) | 1626 (76) | 939 (74) | 687 (79) | Ref | | - |
| Unknown | | 993 (83) | 745 (82) | 248 (87) | 8155 (79) | 5134 (80) | 3021 (78) | - | | - |
| Gram stain | |  |  |  |  |  |  |  | |  |
| Gram-pos cocci | | 154 (26) | 151 (34) | 3 (2) | 186 (4) | 116 (5) | 70 (4) | 1.02 (0.75-1.39) | | 0.916 |
| Gram-neg bacilli/coccobacilli | | 73 (12) | 53 (12) | 20 (13) | 472 (11) | 252 (10) | 220 (12) | **0.68 (0.54-0.86)** | | **0.001** |
| Gram-neg cocci | | 23 (4) | 21 (5) | 2 (1) | 77 (2) | 25 (1) | 52 (3) | **0.49 (0.30-0.81)** | | **0.005** |
| Neg/Nothing of significance | | 341 (58) | 212 (48) | 129 (84) | 3604 (83) | 2166 (84) | 1438 (81) | Ref | | - |
| Unknown | | 604 (51) | 473 (52) | 131 (46) | 5960 (58) | 3846 (60) | 2114 (54) | - | | - |

^a^ HNS = multiplex PCR which detects *H. influenzae*, *N. meningitidis* and *S. pneumoniae*

^b^ Adjusted odds ratio, controlling for geographic location (country), comparing characteristics by RNase P PCR result [(RNase P positive (C_t_<36) vs RNase P negative (C_t_>36)]

^c^ Denominators used to calculate % for each characteristic excluded those samples with data unknown.

^d^ Hi – *H. influenzae*, Sp – *S. pneumoniae*, Nm – *N. meningitidis*
